# Metagenomic Insights into the RDX-Degrading Potential of the Ovine Rumen Microbiome

**DOI:** 10.1371/journal.pone.0110505

**Published:** 2014-11-10

**Authors:** Robert W. Li, Juan Gabriel Giarrizzo, Sitao Wu, Weizhong Li, Jennifer M. Duringer, A. Morrie Craig

**Affiliations:** 1 United States Department of Agriculture, Agriculture Research Service, Animal Genomics and Improvement Laboratory, Beltsville, Maryland, United States of America; 2 College of Veterinary Medicine, Oregon State University, Corvallis, Oregon, United States of America; 3 Center for Research in Biological Systems, University of California San Diego, La Jolla, California, United States of America; 4 Department of Environmental & Molecular Toxicology, Oregon State University, Corvallis, Oregon, United States of America; Miami University, United States of America

## Abstract

The manufacturing processes of royal demolition explosive (RDX), or hexahydro-1,3,5-trinitro-1,3,5-triazine, have resulted in serious water contamination. As a potential carcinogen, RDX can cause a broad range of harmful effects to humans and animals. The ovine rumen is capable of rapid degradation of nitroaromatic compounds, including RDX. While ruminal RDX-degrading bacteria have been identified, the genes and pathways responsible for RDX degradation in the rumen have yet to be characterized. In this study, we characterized the metabolic potential of the ovine rumen using metagenomic approaches. Sequences homologous to at least five RDX-degrading genes cloned from environmental samples (*diaA*, *xenA*, *xenB*, *xplA*, and *xplB*) were present in the ovine rumen microbiome. Among them, *diaA* was the most abundant, likely reflective of the predominance of the genus *Clostridium* in the ovine rumen. At least ten genera known to harbor RDX-degrading microorganisms were detectable. Metagenomic sequences were also annotated using public databases, such as Pfam, COG, and KEGG. Five of the six Pfam protein families known to be responsible for RDX degradation in environmental samples were identified in the ovine rumen. However, increased substrate availability did not appear to enhance the proliferation of RDX-degrading bacteria and alter the microbial composition of the ovine rumen. This implies that the RDX-degrading capacity of the ovine rumen microbiome is likely regulated at the transcription level. Our results provide metagenomic insights into the RDX-degrading potential of the ovine rumen, and they will facilitate the development of novel and economic bioremediation strategies.

## Introduction

Hexahydro-1,3,5-trinitro-1,3,5-triazine, also known as royal demolition explosive (RDX), has replaced trinitrotoluene (TNT) over the past few decades as the primary nitroaromatic compound used in explosives [1]. RDX is widely used in US military munitions, and it is present in more than 4,000 military items, from large bombs to very small igniters. However, its manufacturing processes have generated significant amounts of RDX-contaminated wastewater. RDX is a potential carcinogen that causes a broad range of negative effects in humans and animals, including convulsions, loss of consciousness, vomiting, and skin lesions [2]. Moreover, RDX in contaminated soil is mobile, and it can seep into surface water and even groundwater [3]. The unique chemical structure of RDX makes it recalcitrant to chemical and biological degradation, and therefore it is very difficult to eliminate from contaminated environments [4].

It has long been known that numerous microorganisms, such as bacteria and fungi, are able to degrade RDX [5]. A variety of biochemical pathways responsible for RDX degradation in soil and groundwater under aerobic or anaerobic conditions have been proposed [5]. These pathways include denitration, direct enzymatic cleavage [6], reduction by biogenic Fe(II) [7], and mineralization [8]. Under anaerobic conditions, mechanisms via reduction followed by ring cleavage or via direct ring cleavage have been proposed [6], [9]–[11]. The sequential formation of nitroso products, including hexahydro-1-nitroso-3,5-dinitro-1,3,5-triazine (MNX), hexahydro-1,3-dinitroso-5-nitro-1,3,5-triazine (DNX), and hexahydro-1,3,5-trinitroso-1,3,5-triazine (TNX), was detectable as one route, while direct ring cleavage leading to the formation of methylenedinitramine (MEDINA) and bis(hydroxymethyl)nitramine (BHNA) was observed as a second route.

Over the past few decades, efforts have been made to identify genes/enzymes or microbial strains capable of the biochemical reactions that contribute to RDX biodegradation. For example, diaphorase, an enzyme from the anaerobic bacterium *Clostridium kluyveri* (originally isolated from mud) has been known to catalyze RDX biotransformation [12]. Diaphorase, consisting of two important domains, a flavin-reductase domain and a rubredoxin-like domain, is oxygen sensitive and catalyzes RDX degradation via denitration, as nitroso intermediates such as MNX, DNX, and TNX are not detected. The gene encoding diaphorase, *diaA*, has been cloned [13]. In addition, oxygen-insensitive (type I) NADPH nitroreductases (*nsfI*) cloned from many bacteria, particularly enterobacteria, such as *Enterobacter cloacae* and *Morganella morganii*, are also able to degrade RDX [14]. Two flavin mononucleotide-containing oxidoreductases, or xenobiotic reductase, genes *xenA* and *xenB*, have been cloned from *Pseudomonas putida* and *P. fluorescens*, respectively [15]. Pure culture of the *Pseudomonas* strains harboring these two genes demonstrated that *xenB* was able to degrade RDX faster than *xenA* under anaerobic conditions [16]; in addition, *xenB* displayed a broader substrate specificity than *xenA*. The activities of both enzymes are always higher when degrading RDX under anaerobic conditions than under aerobic conditions. The date, the best known enzymes for RDX biotransformation are found in a unique cytochrome p450 enzyme pair [17]. This system, consisting of fused flavodoxin–cytochrome p450 *xplA* and its partnering flavodoxin, reductase *xplB,* is able to denitrate RDX reductively under both aerobic and anaerobic conditions. These two genes were originally cloned from the genome of *Rhodococcus rhodochrous* [18]. In *R. rhodochrous* strain 11Y, *xplA* and *xplB* are both transcribed constitutively. Furthermore, RDX degradation was positively correlated with *xplA* expression at the mRNA level; both *xplA* and *xplB* were strongly upregulated by the presence of RDX [19], [20]. Further studies in other *Rhodococcus* strains suggest that this system is likely the key enzyme responsible for RDX biotransformation in the genus *Rhodococcus* [21]. It has been shown that in an *in vivo* system, the two co-expressed enzymes are able to degrade RDX more efficiently than either one alone. Transgenic plants expressing both *xplA* and *xplB* were able to degrade saturating levels of RDX in liquid cultures and soil leachate [22]. *xplA* and *xplB* genes are encoded by exochromosomal mobile elements [23], and they are present in microbes from at least three genera commonly found in soil and groundwater – *Rhodococcus*, *Gordonia*, and *Williamsia* [9]. Lateral gene transfer may contribute to their wide geographical and phylogenetic distribution [23]. The global distribution of RDX-degrading bacteria containing *xplA* gene homologs suggests that denitration may represent a fundamental RDX degradation pathway [24].

The rumen is efficient at biotransforming nitroaromatic compounds, including TNT, RDX, and High Melting eXplosive (HMX) [10], [25], [26]. Compared to other microbial ecosystems, such as soil and groundwater, the rumen is capable of rapid degradation of RDX, in an *in situ* manner. Greater than 98% of RDX is biotransformed from its initial concentration of 25 µg/µl within four hours of incubation. By the end of an eight-hour incubation, RDX cannot be detected using traditional detection methods, high-performance liquid chromatography (HPLC) and LC-MS/MS [27]. Thus far, the unique anaerobic environment of the rumen, with its high redox potential, appears to be highly efficient at biodegrading RDX, and RDX-degrading bacteria have been identified from the rumen [11], [27]. However, genes and metabolic pathways leading to RDX biodegradation in the ovine rumen have yet to be elucidated. In this study, the relative abundance of genes associated with RDX degradation in the rumen was systematically surveyed. In addition, the metabolic potential of RDX degradation in the ovine rumen was characterized using metagenomic approaches.

## Materials and Methods

### Animals

Five one-year-old male sheep were used in this study. After weaning, the sheep were grass-fed *ad libitum* until slaughter. Rumen fluid samples were collected in a local commercial slaughter house (Mohawk Meats, Springfield, OR, USA). The Food Safety and Inspection Service directives for humane handling and slaughtering of livestock under the jurisdiction of the United States Department of Agriculture (USDA) were strictly followed during animal husbandry and slaughtering. All animal procedures were carried out in accordance with the protocols approved by the USDA Beltsville Animal Care and Use Committee (Protocol #12-025). Whole rumen samples were dissected from the animals after slaughter. A sterile aluminum cannula, attached to a 50 ml syringe, was inserted into the rumen after performing an incision with a sterile scalpel to collect rumen fluid. Approximately 100 ml of whole rumen fluid (WRF) were collected, samples were placed in a 39°C thermos and immediately transported to the lab for further processing.

### RDX degradation experiments

WRF was supplemented with RDX under anaerobic conditions to a final concentration of 40 µg/µl. Deionized water instead of RDX was added to WRF to serve as time-matched basal controls. Approximately 20 ml of enriched WRF were placed into glass tubes and sealed with butyl rubber stoppers. The solution was incubated at 39°C on a tray shaker under anaerobic conditions; samples were collected by sterile puncture of the butyl rubber stoppers at different time points up to 240 min. The samples were immediately extracted using a liquid–liquid chemical extraction technique as described in [28]. Briefly, the collected samples (2 ml) were centrifuged at 10,000 *g* for 5 min. The supernatant (300 µl) was distributed into glass vials and mixed with 300 µl of a 0.34 M ammonium hydroxide solution. The samples were then transferred to 2 ml screw-cap microcentrifuge tubes, mixed with 1 ml of hexane:ethyl acetate (1∶1) solution, and shaken vigorously for 10s. Approximately 800 µl of the organic phase were transferred to a separate tube. This procedure was repeated three times; the organic phase was then pooled to a final volume of 2.4 ml. The samples were dried under a constant stream of N_2_ and stored at −20°C until HPLC–UV [26] and LC–MS/MS analysis [11].

### Metagenomic DNA extraction and sequencing

Metagenomic DNA extraction was executed using a QIAamp DNA stool kit (Qiagen, Valencia, CA) with modifications as previously described [29], [30]. DNA integrity and concentration were estimated using a Bioanalyzer 2100 system (Agilent, Palo Alto, CA). Approximately 1.0 µg of high-quality DNA was processed using an Illumina TruSeq DNA sample prep kit following the manufacturer’s instructions (Illumina, San Diego, CA). Final individual libraries were validated, pooled based on their respective 6-bp adaptors, and sequenced at 2×100 bp/sequence read (pair-end) using an Illumina HiSeq 2000 sequencer. Approximately 52,161,704.92±14,640,816.82 (mean ± SD) whole genome shotgun (WGS) pair-ended sequence reads per sample were generated for this study. The raw reads were deposited in the MG-RAST server (http://metagenomics.anl.gov/; Accession numbers 4552886.3 to 4552888.3, 4552952.3 to 4552965.3, 4552985.3 to 4552986.3, 4553942.3 to 4553951.3, and 4554052.3 to 4554061.3).

The 16S rRNA gene amplicons were directly generated from metagenomic DNA samples using PAGE-purified oligos containing Illumina-platform compatible adaptors and PCR primer sequences targeting the hypervariable V3–V4 regions of the 16 rRNA gene (341/357F and 805R, Primer names S-D-Bact-0341-b-S-17 and S-D-Bact-0785-a-A-21, respectively). Briefly. the 16S amplicons were generated from 20 ng of metagenomic DNA using 2.5 units of AccuPrime Taq DNA Polymerase High Fidelity (Invitrogen, Carlsbad, CA) in a 50-µl reaction buffer containing 200 nM primers, 200 nM dNTP, 60 mM Tris-SO_4_, 18 mM (NH4)_2_ SO_4_, 2.0 mM MgSO_4_, 1% glycerol, and 100 ng/ul bovine serum albumin (New England BioLabs, Ipswich, MA). PCR was performed using the following cycling profile: Initial denaturing at 95°C for two min followed by 22 cycles of 95°C 30 s, 50°C 30 sec, and 72°C 60 sec. Amplicons were then purified using Agencourt AMPure XP beads (Beckman Coulter Genomics, Danvers, MA) and quantified using a BioAnalyzer high-sensitivity DNA chip kit (Agilent). Amplicons from individual samples were pooled at equal molar ratios and directly sequenced at 2×250 bp/sequence read (pair-end) using an Illumina MiSeq sequencer.

### Data analysis and statistics

Raw sequence reads were first pre-processed for quality control (QC). Reads of host origin were removed using Bowtie and FR-HIT [31]. The bovine (*Bos taurus* UMD v3.1) and sheep genomes (Oar v2.0) were used to filter out the sequence reads of host origin. A maximum number of four mismatches was allowed. SolexaQA (v2.2), a Perl-based software package for calculating quality statistics from FASTQ files generated by Illumina sequencers [32], was used to trim low-quality reads. Low-quality reads were also removed based on an error probability model (error rate cutoff = 0.05). After these steps, only pair-end reads of the same length (≥80 bp) were retained for further analysis.

After the QC steps, the WGS sequences were assembled *de novo* for downstream analyses; assembly generally improves functional annotation. MetaVelvet, a recently developed *de novo* short-read assembler designed specifically to handle metagenomic datasets [33], was used to assemble the processed reads (parameters used: Kmer = 55; insert size = 400 bp; and minimum contig length cutoff: 100 bp). In a test run, MetaVelvet was able to generate longer contigs of higher quality than other popular short-read assemblers, such as SOAP*denovo* and Velvet, resulting in an increased number of predicted genes.

Open reading frames (ORF) were predicted from contigs greater than 200 bp using FragGeneScan [34], a gene-calling program that combines sequencing error models and codon usages in a hidden Markov model to improve the prediction of protein-coding regions in short reads. Predicted ORFs were further annotated against the Pfam database [35], a widely used database for protein family analysis that includes more than 14,800 annotated protein families in its latest release (v27.0). These families are also organized into groupings of related families (clans), based on similarity of sequences and structures. Pfam seed alignments were downloaded and a database of core profile HMMs was compiled to annotate predicted proteins using the HMMSCAN software package (v3.0, E value cutoff = 0.001). Gene Ontology (GO) was then extracted from these protein families using the Pfam2GO program [36].

The raw sequence reads and contigs generated by MetaVelvet were uploaded into an MG-RAST server [37] and analyzed following the MG-RAST pipeline (v3.0), including quality filtering, dereplication to remove possible sequencing artifacts, and removal of host contaminants using the default settings. ORFs were annotated against the COG database [38] to classify their functional categories. Metabolic pathways were analyzed using pathway databases, such as KEGG. The metagenomic features, such as Pfam and COG profiles and metabolic pathway data, of the samples from the various treatment groups were analyzed using the statistical package MetaStats [39].

ORFs were also predicted from unassembled individual sequence reads using FragGeneScan as described above. Amino acid sequences of these ORFs (query) were analyzed using BLASTP against the sequences of six genes known to be involved in RDX degradation–*diaA* (Accession #YP_001396175.1), *xenA* and *xenB* (AF154061 and AF154062), *xplA* and *xplB* (AF449421), and *nsf1* (M63808.1). The cutoff parameters used were 70% sequence identity with 70% minimal alignment length to input query sequences. Hit counts (abundance) were then analyzed using a modified *t*-test with permutation.

The four-base random sequences (NNNN) in modified Illumina sequencing adaptors to increase sequence diversity were first removed. Paired-end raw reads were then scanned for the presence of primer sequences. The reads without primer sequences were discarded. The pair-end reads were then merged to form contig sequences. A three-base mismatch was tolerated in the overlapped regions. Approximately 95% of processed pair-end reads after quality control procedures can be merged. The mean numbers of consensus contig sequences per sample were 89,541.00±10,903.54 (± sd). Resultant contig sequences were analyzed using RDP Classifier from the Ribosomal Database Project (https://pyro.cme.msu.edu/index.jsp) at a 80% confidence threshold for taxonomic classification. Taxonomy-independent clustering methods, CD-HIT-OTU and RDPipeline, were used to cluster the contigs to generate operational taxonomic units (OTU). Sequence count data were then normalized and analyzed using a modified *t*-test. The consensus sequences of clusters identified using CD-HIT-OTU were then annotated against the GreenGene database.

## Results

### Abundance of known RDX-degrading gene homologues in the ovine rumen

**Figure 1:**
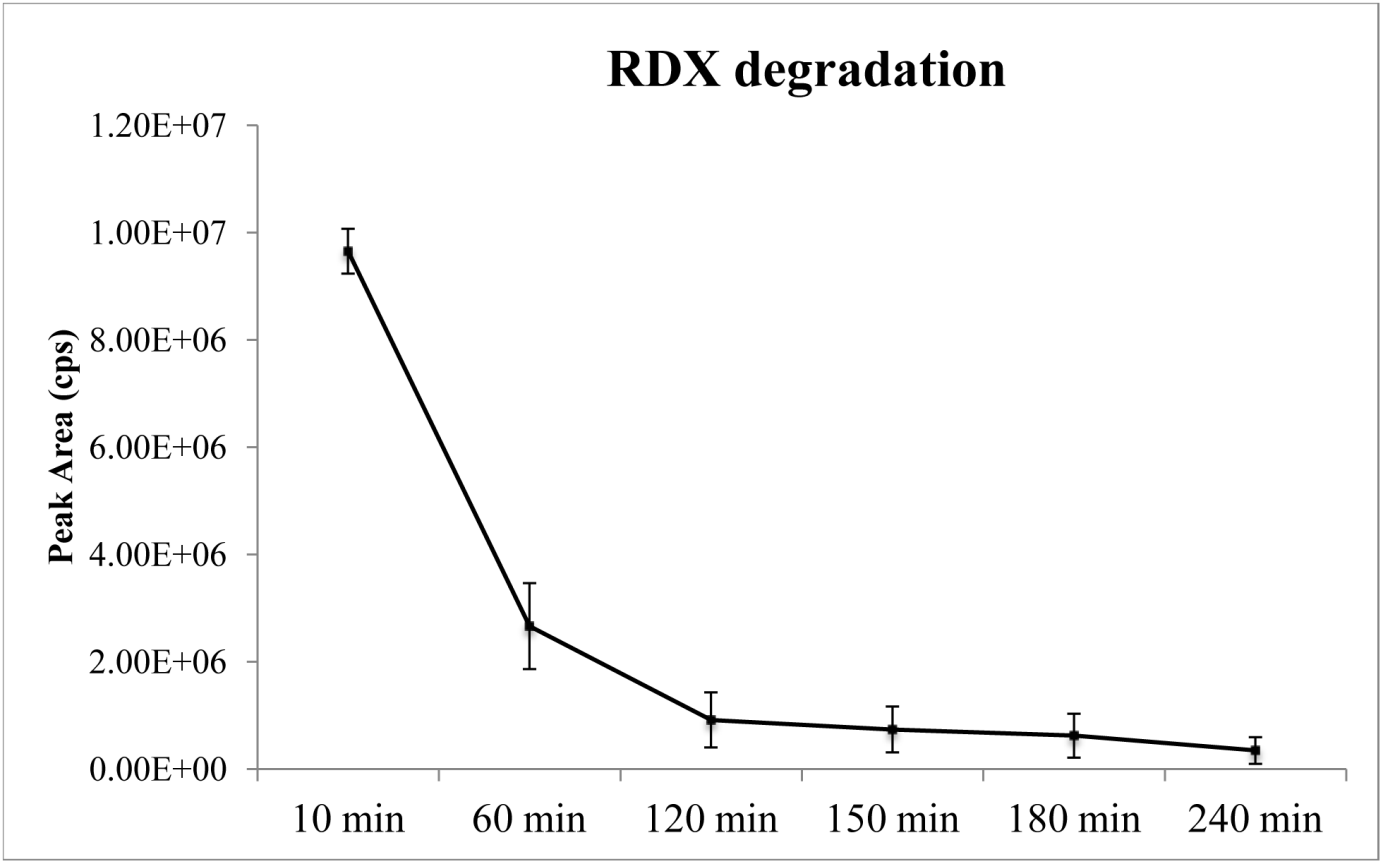
RDX degradation by the ovine rumen microbiome over time. Error bars represent SE (*N* = 5).

A time curve of RDX degradation by the ovine rumen microbiome in an *ex vivo* model is depicted in Figure 1. A one-hour incubation in WRF resulted in a reduction of approximately 72% of the initial RDX concentration (40 µg/µL). By the end of a four-hour incubation, only approximately 3. 6% of the initial RDX remained. The rapid degradation of RDX by the ovine rumen microbiome is in agreement with previous observations [27]. The rumen microbiome was sampled for metagenomic analysis from four of the six time points tested (10, 60, 150 and 240 min).

At least six genes known to be involved in RDX degradation have been cloned from environmental samples: *diaA*, *xenA* and *xenB*, *xplA* and *xplB*, and *nsf1*. To survey the relative abundance of sequences homologous to these genes, a BLASTP search was conducted. ORFs predicted from unassembled sequences were used in order to have a more accurate estimate of the relative abundance. The sequence homologues of five of the six genes were reliably detectable in the ovine rumen. However, their relative abundance varied tremendously. As shown in Table 1, the *diaA* gene homologues were the most abundant in the ovine rumen, with 49.76±25.87 (mean ± SD) per 10 million input sequences. The *xenA* and *xenB* homologues accounted for 7.58 and 5.22 hits, respectively, per 10 million input sequences. The *xplA* and *xplB* homologues were barely detectable (<1 hit per 10 million reads), while sequences homologous to *nsfI* were not detectable. Furthermore, the relative abundance of these homologues remained stable during RDX incubation (Table 1).

**Table 1:** The relative abundance of genes involved in RDX degradation in the ovine rumen.

Gene	Treatment	Incubation (minutes)*			
		10	60	150	240
*diaA*					
	−RDX	49.76±25.87	56.50±20.91	55.70±23.10	54.70±13.70
	+RDX	44.49±7.52	41.86±9.25	52.70±15.14	38.68±14.83
*xenA*					
	−RDX	7.58±3.92	8.04±5.65	8.87±6.96	6.57±4.16
	+RDX	4.48±2.32	6.25±3.66	7.59±4.25	5.52±5.52
*xenB*					
	−RDX	5.22±3.57	4.47±1.26	4.74±1.66	5.34±3.63
	+RDX	4.72±1.52	4.64±1.06	3.91±1.88	3.90±1.97

^*^The number denotes BLASTP hits per 10 million input sequences (±SD). The minimum % identity cutoff was 70%; the minimum alignment length was 21 amino acids.

### Protein repertoire of the ovine rumen microbiome

**Figure 2:**
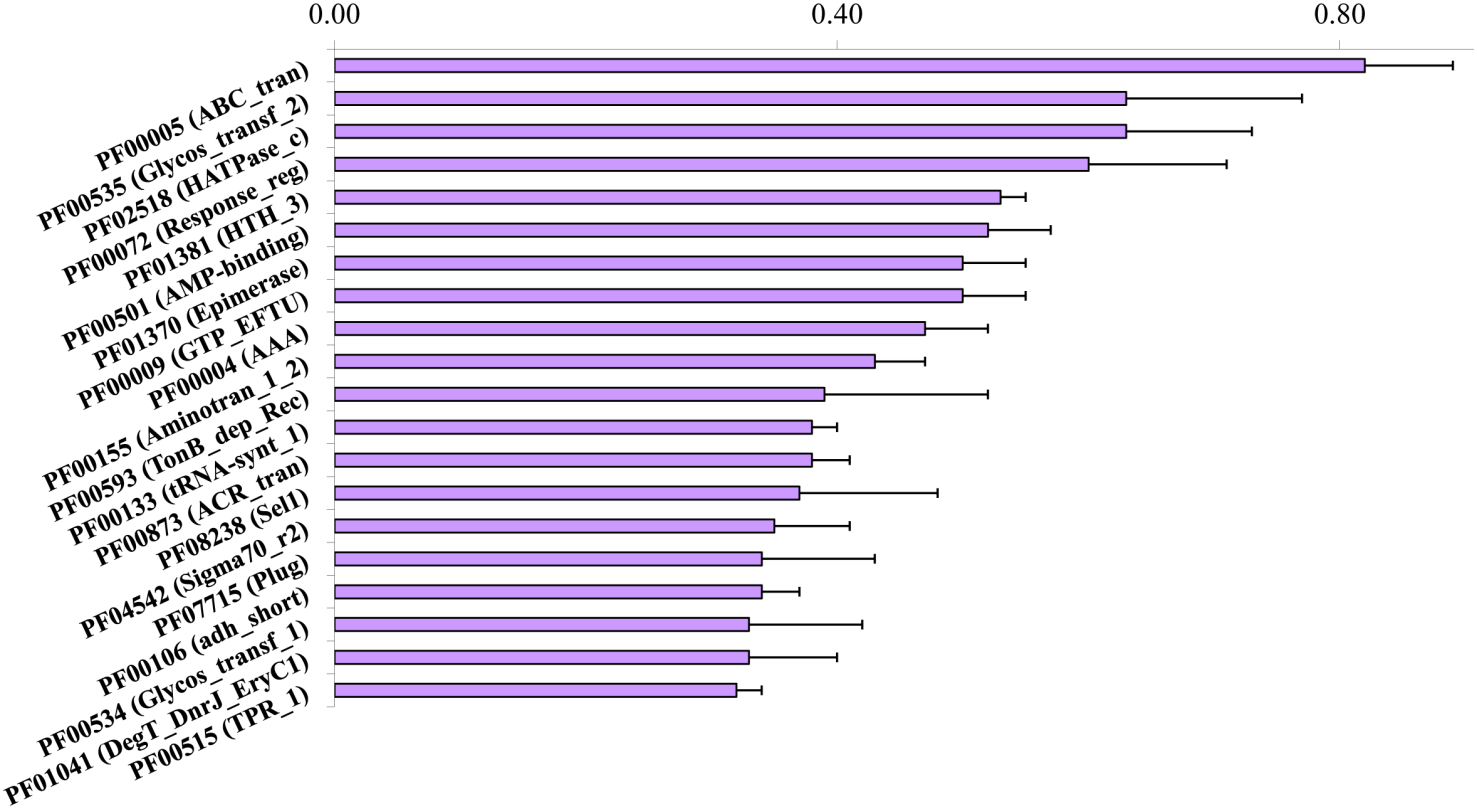
The 20 most common Pfam protein families in the ovine rumen microbiome. Error bars represent SD (*N* = 5). X-axis: relative abundance (%).

A *de novo* assembly of processed sequence reads using MetaVelvet resulted in 142,061 contigs with a mean *N*50 = 715 bp (±337.32; SD) per sample. Approximately 175,375 ORFs were predicted from these contigs using FragGeneScan. The ORFs were annotated against the Pfam database;. the mean number of Pfam protein families identified in the ovine rumen microbiome at the basal control level was 2,765±57 (Table S1). The 20 most common Pfam families are shown in Figure 2. The 100 most common Pfam families accounted for approximately 25.5% of the ORFs assigned to all Pfam families, and they may contribute to the basic function of the ovine rumen microbiome. The most common Pfam family was ABC transporter (PF00005, 0.82%), as in many other microbial ecosystems including the bovine rumen [29] and the porcine colon microbiome [40], [41]. Glycosyl transferase family 2 (PF00535) was the second most abundant family with 0.63% of all hits. In addition, 29 Pfam families associated with glycosyl hydrolase (GH) activities were detected in each of the ovine rumen samples tested. GH43 (PF04616), GH3 (PF00933), GH97 (PF10566), GH2 (PF02836 and PF02837), and GH31 (PF01055) were among the most abundant GH families, suggesting that the ovine rumen possesses sufficient lignocellulolytic capacity. However, GH5 and GH15 were very rare in these samples. GH5, originally known as cellulase family A, includes a wide range of enzymes acting on β-linked carbohydrates (oligo- and polysaccharides), as well as glycoconjugates. GH15 enzymes, on the other hand, carry out inverting reactions and possess three known enzymatic activities: glucoamylase, glucodextranase, and α,α-trehalase. However, the biological relevance of the underrepresentation of these two GH families in the ovine rumen remains unknown.

Approximately 900 GO terms associated with the Pfam protein families were identified. The most common GO Molecular Function terms included ATP binding (6.61%), catalytic activity (3.20%), DNA binding (3.13%), cxidoreductase activity (2.30%), GTP binding (1.80%), hydrolase activity (1.59%), RNA binding (1.29%), and transporter activity (1.19%). Similarly, the five most common GO Biological Process terms were metabolic process (2.93%), carbohydrate metabolic process (2.18%), oxidation-reduction process (1.88%), biosynthesis process (1.79%), and proteolysis (1.69%). Intriguingly, at least six GO Molecular Function terms related to RDX degradation were detected in the ovine rumen: GO:0016491 (oxidoreductase activity), GO:0009055 (electron carrier activity), GO:0010181 (FMN binding), GO:0005506 (iron ion binding), GO:0016705 (oxidoreductase activity, acting on paired donors), and GO:0020037 (heme binding), as well as one Biological Process term, GO:0055114 (oxidation-reduction process). Furthermore, oxidoreductase activity (GO:0016491) and oxidation-reduction process (GO:0055114) were among the five most abundant GO terms (Table 2).

**Table 2:** The relative abundance of Pfam protein families and Gene Ontology (GO) involved in RDX degradation remained unchanged in the ovine rumen microbiome during RDX incubation.

Pfam	Treatment	Incubation (minutes)*			
		10	60	150	240
PF00067 (p450)					
	−RDX	0.00±0.00	0.00±0.00	0.00±0.00	0.00±0.00
	+RDX	0.00±0.00	0.00±0.00	0.00±0.00	3.39±4.66
PF00258 (Flavodoxin_1)					
	−RDX	2.93±0.18	2.89±0.95	3.71±1.59	2.37±1.50
	+RDX	3.41±2.33	2.69±1.74	2.96±0.28	3.22±0.89
PF00301 (Rubredoxin)					
	−RDX	3.91±0.91	3.90±0.81	3.77±0.79	4.61±3.36
	+RDX	4.83±1.05	2.31±1.65	3.71±0.61	3.42±1.88
PF00724 (Oxidored_FMN)					
	−RDX	2.33±0.91	2.17±1.39	2.21±1.61	6.00±6.03
	+RDX	2.68±1.91	4.15±2.31	3.08±1.81	1.63±0.89
PF01613 (Flavin_Reduct)					
	−RDX	5.39±0.98	5.02±0.62	5.83±0.80	5.89±2.65
	+RDX	5.59±2.07	4.83±1.91	4.81±1.95	3.23±0.88
GO:0016491 (Oxidoreductase activity)					
	−RDX	2.30±0.03	2.31±0.05	2.52±0.08	2.32±0.09
	+RDX	2.37±0.07	2.08±0.21	2.46±0.04	2.29±0.16
GO:0055114 (Oxidation-reduction process)					
	−RDX	1.88±0.04	1.91±0.05	1.96±0.07	1.83±0.06
	+RDX	1.86±0.06	1.70±0.18	2.07±0.06	1.96±0.12

Note: *the number (mean±SE) denotes normalized counts of ORFs positively assigned to this Pfam (per 10,000 ORFs assigned to any Pfam families or percentage of hits assigned to the GO term.

The ovine rumen protein repertoire was also assessed using COG database annotation. The mean number of COGs identified per sample was 1,816 (±34.8; SD), while 1,272 COGs were shared by all rumen samples (Table S2). The 1,272 COGs accounted for 92.5% of the sequences assigned, and the 100 most abundant COGs represented approximately 27% of the sequences. The five most abundant COGs were predicted ATPase (AAA+ superfamily) (COG1373, 0.78%), signal transduction histidine kinase (COG0642, 0.71%), β-galactosidase/β-glucuronidase (COG3250, 0.60%), glycosyltransferases involved in cell wall biogenesis (COG0463, 0.59%), and translation elongation factors (GTPases) (COG0480, 0.57%). Similarly, two COGs, pyruvate:ferredoxin oxidoreductase and related 2-oxoacid:ferredoxin oxidoreductases, γ (COG1014) and α (COG0674) subunits, were among the ten most abundant COGs. These two COGs were likely related to RDX degradation.

### Effects of RDX exposure on the ovine rumen microbiome

**Figure 3:**
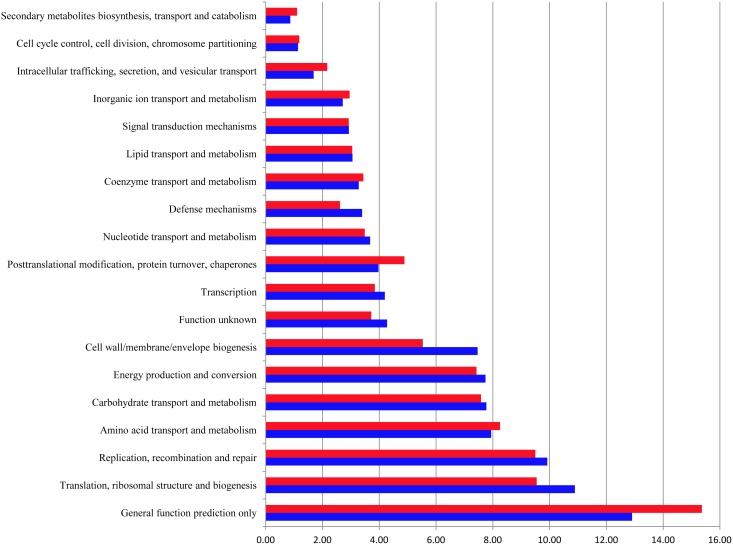
Functional categories affected by RDX exposure to the ovine rumen microbiome, annotated using the COG database. Blue: control (*N* = 5); Red: RDX exposure (*N* = 5).

Possible changes in ovine rumen metagenomic parameters induced during RDX degradation were first assessed at the functional category level using the COG database. Among the 26 categories identified, Translation, ribosomal structure and biogenesis, Replication, recombination and repair, Amino acid transport and metabolism, and Carbohydrate transport and metabolism were the most abundant. As shown in Figure 3, the percentage of hits assigned to these 26 categories remained unchanged (false discovery rate FDR>0.05) during RDX degradation at each of the four time points tested. Moreover, at any given time point, none of the individual COGs displayed a statistically significant difference in relative abundance between the two treatment groups.

**Figure 4:**
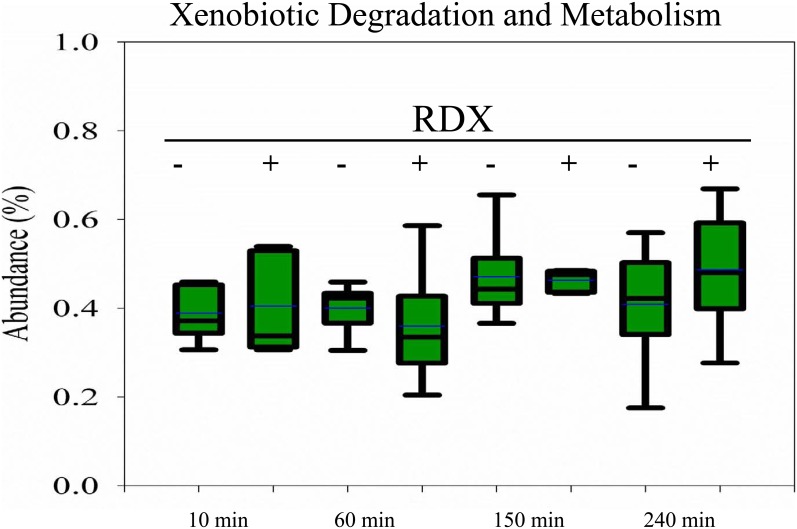
Relative abundance of sequences annotated to the KEGG Xenobiotic Biodegradation and Metabolism term during exposure of the ovine rumen to RDX over four hours. Boxes denote the inter-quartile range between the first and third quartiles (25 and 75%, respectively). (–): time-matched WRF control without RDX exposure. (+): WRF incubated with 40 µg/µl RDX.

The KEGG Orthology (KO) analysis suggested that 107 KO pathways were shared by all of the rumen samples. These pathways accounted for >99% of the hits assigned to all pathways, and they may contribute to the basic metabolic function of the ovine rumen. No significant alterations to the relative abundance of these pathways were observed during RDX degradation at 150 and 240 minutes (Table 3). At higher classification levels, neither the number of hits assigned to Metabolism nor one of its sub-categories, Xenobiotics biodegradation and metabolism, was significantly changed during RDX degradation from 10 to 240 minutes (Figure 4, FDR>0.05).

**Table 3:** The relative abundance of 40 KEGG Orthology (KO) pathways with (+) and without (–) RDX incubation in the ovine rumen.

		150 min		240 min	
KO_id	Pathway	–RDX	+RDX	–RDX	+RDX
ko00970	Aminoacyl-tRNAbiosynthesis	6.87±1.19	7.18±0.71	6.78±0.82	6.07±1.50
ko03010	Ribosome	6.68±1.46	6.44±1.69	7.65±2.73	5.31±1.76
ko00250	Alanine, aspartate andglutamate metabolism	4.68±0.53	4.87±0.58	4.38±0.81	3.88±1.30
ko00230	Purine metabolism	3.74±0.45	3.74±0.19	3.56±0.28	3.09±0.72
ko00260	Glycine, serine and threoninemetabolism	3.56±0.27	3.49±0.35	3.34±0.48	2.96±0.93
ko02010	ABC transporters	3.51±0.40	3.65±1.13	4.30±1.92	3.53±2.43
ko00190	Oxidative phosphorylation	2.66±0.11	2.66±0.21	2.74±0.54	2.18±0.69
ko03020	RNA polymerase	2.46±0.32	2.58±0.19	2.57±0.61	2.35±0.62
ko03030	DNA replication	2.43±0.08	2.50±0.25	2.80±0.45	2.15±0.59
ko03018	RNA degradation	2.35±0.42	2.55±0.26	2.75±0.43	2.47±0.28
ko03070	Bacterial secretion system	2.20±0.35	2.30±0.14	2.36±0.41	1.87±0.73
ko00240	Pyrimidine metabolism	2.06±0.37	1.95±0.07	1.88±0.25	1.61±0.49
ko00010	Glycolysis/Gluconeogenesis	2.06±0.20	2.19±0.35	1.94±0.38	1.99±0.72
ko00270	Cysteine and methioninemetabolism	1.95±0.27	1.97±0.28	1.80±0.20	1.59±0.45
ko00040	Pentose and glucuronateinterconversions	1.90±0.43	1.65±0.55	1.48±0.80	1.35±0.54
ko00330	Arginine and proline metabolism	1.89±0.19	1.77±0.16	1.66±0.39	1.64±0.56
ko04112	Cell cycle - Caulobacter	1.86±0.21	1.85±0.07	1.97±0.37	1.37±0.37
ko02020	Two-component system	1.84±0.05	1.71±0.28	1.68±0.30	1.49±0.45
ko03440	Homologous recombination	1.81±0.27	1.62±0.27	1.94±0.56	1.42±0.54
ko00051	Fructose and mannose metabolism	1.80±0.13	1.79±0.04	1.53±0.35	1.32±0.29
ko00052	Galactose metabolism	1.69±0.10	1.63±0.36	1.51±0.35	1.28±0.48
ko00520	Amino sugar and nucleotide sugar metabolism	1.67±0.25	1.54±0.37	1.42±0.30	1.34±0.51
ko00550	Peptidoglycan biosynthesis	1.63±0.15	1.68±0.20	1.73±0.26	1.24±0.40
ko00500	Starch and sucrose metabolism	1.61±0.17	1.45±0.13	1.41±0.16	1.28±0.35
ko03420	Nucleotide excision repair	1.59±0.47	1.68±0.36	1.65±0.22	1.28±0.34
ko00300	Lysine biosynthesis	1.49±0.12	1.38±0.08	1.31±0.27	1.16±0.41
ko00020	Citrate cycle (TCA cycle)	1.46±0.08	1.50±0.38	1.41±0.33	1.18±0.47
ko00620	Pyruvate metabolism	1.40±0.27	1.47±0.25	1.26±0.14	1.24±0.53
ko00340	Histidine metabolism	1.34±0.16	1.21±0.17	1.20±0.24	0.98±0.35
ko00400	Phe, tyr and try biosynthesis	1.19±0.35	1.19±0.32	0.97±0.47	0.75±0.36
ko00030	Pentose phosphate pathway	1.18±0.16	1.24±0.06	1.11±0.14	0.94±0.24
ko00280	Valine, leucine and isoleucinedegradation	1.14±0.34	1.22±0.16	1.32±0.25	1.11±0.34
ko00061	Fatty acid biosynthesis	1.13±0.14	1.24±0.11	1.10±0.33	0.99±0.26
ko00540	Lipopolysaccharide biosynthesis	1.10±0.13	1.10±0.12	1.21±0.28	0.80±0.24
ko00290	Valine, leucine and isoleucinebiosynthesis	1.08±0.23	1.15±0.30	0.88±0.50	0.86±0.33
ko00900	Terpenoid backbone biosynthesis	1.07±0.07	1.06±0.11	1.17±0.11	0.88±0.28
ko00860	Porphyrin and chlorophyllmetabolism	1.05±0.36	1.04±0.38	0.78±0.39	0.86±0.17
ko00940	Phenylpropanoid biosynthesis	1.03±0.30	0.99±0.47	0.76±0.66	0.73±0.52
ko00521	Streptomycin biosynthesis	0.99±0.14	1.02±0.12	1.13±0.23	0.83±0.31
ko00760	Nicotinate and nicotinamidemetabolism	0.89±0.16	0.96±0.04	1.03±0.26	0.85±0.29

Eight GO terms and seven Pfam protein families associated with six known RDX-degrading genes were retrieved using the Pfam database. Seven of the eight GOs and six of the seven Pfam families were detected in the ovine rumen. While the GO terms associated with RDX metabolism, such as oxidoreductase activity (GO:0016491) and oxidation-reduction process (GO:0055114), were among the most abundant GO terms in the ovine rumen, none of the seven GOs displayed significant changes following RDX degradation. Similarly, no statistically significant changes in the abundance of these Pfam protein families were observed during RDX degradation (Table 2).

## Discussion

The rumen microbiome is known to play a critical role in the normal physiology and nutrition of ruminants [29], [30]. It is also well known that rumen microorganisms are important in detoxifying plant secondary metabolites and xenobiotic compounds. Several studies have attempted to survey the microbial composition of the ovine rumen microbiome using culture-independent, 16S rRNA gene-based molecular tools [42]–[45]. However, little is known about the genes and pathways responsible for RDX degradation in the ovine rumen.

Much of our knowledge of RDX metabolism is derived from studies using RDX-degrading bacterial and fungal strains of environmental origin in pure culture. At least 23 strains have been isolated from environmental samples to date, under either aerobic or anaerobic conditions [5]. The cluster analysis of 16S rRNA gene sequences of these isolates suggests that RDX-degrading bacteria are widely distributed and belong to at least six classes, such as Actinobacteria, Clostridia, α-, β-, γ-, and δ-Proteobacteria [5]. Our analysis using the least common ancestor method and the 16S rRNA gene sequences (Table S3) indicated that the sequences homologous to these classes were present in the ovine rumen microbiome. Moreover, Clostridia was the second most abundant class while the remaining five classes were among the 20 most abundant in the ovine rumen. Sequences displaying significant matches to at least ten genera that contain known RDX-degrading species isolated from environmental samples were detected in the ovine rumen, including *Clostridium*, *Pseudomonas*, *Geobacter*, and *Acetobacterium*. A recent study of 24 bacterial isolates of rumen origin demonstrated that all 24 isolates possessed varying RDX-degrading abilities under anaerobic conditions [11]. Compared to their respective basal controls, approximately 50% of those isolates were able to degrade RDX as the sole source of nitrogen, while eight isolates were capable of using RDX as the sole source of carbon, after a 120 h incubation. The isolates belong to ten genera; all of which were detectable in the ovine rumen in our study. *Prevotella* was the most abundant, representing 60.44% of assigned sequences, and *Butyrivibrio* (3.60%), *Clostridium* (2.56%), *Selenomonas* (2.14%), and *Eubacterium* (1.78%) were among the ten most abundant genera in the ovine rumen microbiome. However, only a few strains of rumen origin, such as one strain each of *C. polysaccharolyticum* and *Megasphaera elsdenii* and two strains each of *S. ruminantium*, HD4 and PC18, are capable of utilizing RDX as both nitrogen and carbon sources, in agreement with previous observations from environmental samples [8]. Together, these results suggest that the ovine rumen microbiome harbors sufficient microbial diversity for RDX metabolism.

The detection of nitroso intermediates, such as MNX and TNX, as well as compounds with *m/z* of 193 and 174, suggests that at least two reductive pathways are responsible for RDX degradation in the ovine rumen [11]. However, the enzymes that carry out these biochemical reactions have not been identified. To gain insight into the genes that might be responsible for RDX degradation in the ovine rumen, we conducted a systematic survey of six known RDX-degrading genes cloned from environmental samples (*diaA*, *xenA*, *xenB*, *xplA*, *xplB* and *nsf1*). The number of sequences homologous to *diaA* appeared to be higher than those of *xenA* and *xenB* (Table 1). The gene *diaA* was originally cloned from *Clostridium kluyveri* of mud origin. A novel strain of *C. kluyveri* was recently isolated from the bovine rumen [46]. While the relative abundance of this strain accounts for only a tiny fraction of the rumen bacteria, its population can be significantly higher in diets containing lucerne silage. The genus *Clostridium* was among the five most abundant genera in the ovine rumen, accounting for ∼2.6% of all sequences. Thus, the relatively high abundance of the *diaA* gene might be reflective of the predominance of *Clostridium* species in the ovine rumen. However, the overall abundance of known RDX-degrading genes in the ovine rumen was relatively low, represented by five to 50 sequences per 10 million input sequences. The sequences homologous to *xplA* and *xplB* were rare, but they could be detected reliably in the ovine rumen, while sequences matched to *nsfI* were not observed. *nsfI* is known to work under aerobic conditions; therefore, it was somewhat expected that this gene would not be present in the highly anaerobic environment of the rumen. The *xplA* gene and its reductase partner, *xplB*, are exochromosomal genes encoded by plasmids [20], [23]. A plasmid containing *xplA* and *xplB* genes was recently isolated from a strain of *Gordonia* [20]. Similarities in COG functional category profiles between this plasmid and those in the ovine rumen (Figure 3) were noticeable. While the metagenomic DNA extraction protocol used in this study has been shown to be capable of capturing all genetic materials, it was not specifically designed to optimize plasmid DNA extraction. With this caveat in mind, our results suggest that other unidentified genes are likely to contribute to RDX degradation in the ovine rumen.

Six of the seven Pfam protein families and seven of the eight GO molecular functions and biological processes implicated in RDX degradation in environmental samples were detected in the ovine rumen microbiome. However, the abundance of the individual Pfam families was low. Among the approximately 900 GO terms identified, two related to RDX degradation, GO:0016491 (oxidoreductase activity) and GO:0055114 (oxidation-reduction process), were among the most abundant. It should be noted that these two GO terms cover an oxidation-reduction reaction with a very broad range of substrates and might not be directly related to RDX degradation. Together, these results suggest that additional Pfam protein families and GO molecular functions and biological processes responsible for RDX degradation in the ovine rumen have yet to be identified. In addition, our efforts in metabolic pathway reconstruction identified more than 260 pathways using the KEGG Orthology (KO) system. One hundred and seven pathways were shared by all of the samples tested and accounted for >99% of sequence hits; they likely contribute to the basic function of the ovine rumen microbiome. However, compared to the controls, none of these pathways displayed significant changes in their relative abundance when RDX was added to WRF media at each of the four time points tested (FDR>0.05). The number of sequences assigned to higher functional categories, such as Environmental Adaptation and Xenobiotic Biodegradation and Metabolism, also remained unchanged. Our findings suggest that exposure of the ovine rumen microbiome to RDX for four hours did not appear to induce a significant change in the structure or metabolic potential of the ovine rumen microbiome.

Previous studies have suggested that elevated levels of substrate availability might be associated with increased proliferation of RDX degrading bacteria. For example, a significantly higher number of 16S rRNA gene sequences assigned to the genus *Prevotella* was observed in the ovine rumen after 8 h of incubation compared to 4 h [27]. In groundwater samples, the production of RDX metabolites corresponds to a microbial community shift from one made up of primarily β-Proteobacteria to a community dominated by δ-Proteobacteria [47]. However, in this study, we did not observe any RDX-induced shifts in the microbial composition (Table S4) or metabolic pathways in the ovine rumen microbiome during a short period (4 h) of RDX exposure. One possible explanation is that measurable changes in the microbial community composition or metabolic potential may take time to develop, and a four-hour incubation might not be sufficient to induce any changes. Significant changes induced by RDX in the number of sequences assigned to the genus *Prevotella* did not show up until eight-hour post-incubation in the rumen, when the substrate was no longer available [27]. However, the most likely explanation is that the RDX-degrading capacity of the ovine rumen microbiome is regulated at the transcriptional level. Previously studies have reported that transcripts of the methanogenesis pathway genes are substantially elevated in the ovine rumen with high methane yields [48]. Indeed, transcripts containing *xplA* and *xplB* genes are approximately four-fold higher during bacterial growth on RDX than on ammonium [20], and the magnitude of upregulation of these genes by RDX can be repressed under nitrogen constraints. Our future work will focus on characterizing the over-expressed genes in response to RDX exposure using metatranscriptomic approaches, especially in metabolically enriched microbial populations using stable isotope labeling techniques. A novel bioremediation strategy involving the use of forage plants to uptake and concentrate RDX from contaminated soil followed by grazing and the subsequent degradation of RDX-containing forages by small ruminants has been proposed under the term “phytoruminal bioremediation.” It is conceivable that the identification and characterization of genes and pathways responsible for RDX degradation in the rumen will directly contribute to the success of novel RDX bioremediation approaches.

## Supporting Information

Table S1 **Raw counts of Pfam protein families in the ovine rumen microbiome during RDX exposure.**
(XLSX)

Table S2 **Raw counts of the KEGG Orthology (KO) categories in the ovine rumen microbiome during RDX exposure.**
(XLSX)

Table S3 **The microbial composition of the ovine rumen.** The 16S rRNA gene sequences (V3 to V4 regions) were generated using the MiSeq platform (2×250 bp, pair-end). The mean number of the 16S raw reads (pairs) per sample is 98,159.50 (±11,376.04, sd). After various quality control procedures, such as chimeric removal, 89,541.00 ± 10,903.54 overlapped reads (>430 bp in length) were obtained per sample. These reads were then analyzed using both taxonomy-dependent (RDP Classifier) and taxonomy-independent OTU-calling algorithms, such as CD-HIT-OTU. The results in the table were obtained using the RDP Classifier.
(XLSX)

Table S4 **The family-level microbial composition in the ovine rumen microbiome in response to RDX incubation.** The abundance data (raw counts) were derived from unassembled whole-genome shotgun sequences using the lowest common ancestor method in the MG-RAST pipeline.
(XLSX)
